# A rapid review on the application of common data models in healthcare: Recommendations for data governance and federated learning in artificial intelligence development

**DOI:** 10.1177/20552076251395536

**Published:** 2025-11-11

**Authors:** Hanna von Gerich, Taridzo Chomutare, Ville Kytö, Peter Lundberg, Troels Siggaard, Laura-Maria Peltonen

**Affiliations:** 1 Department of Health and Social Management, 122208 University of Eastern Finland, Kuopio, Finland; 2 Department of Nursing Science, 8058 University of Turku, Turku, Finland; 3 380763 Norwegian Centre for E-health Research, Tromsø, Norway; 4 Heart Center, 60652 Turku University Hospital and University of Turku, Research Services, Wellbeing Services County of Southwest Finland, Turku, Finland; 5 Clinical Department of Medical Radiation Physics, 4566 Region Östergötland, Linköping, Sweden; 6 Clinical Department of Radiology in Linköping, Region Östergötland, Linköping, Sweden; 7 Center for Medicine Imaging and Visualization Science (CMIV), Linköping University, Linköping, Sweden; 8 Department of Health, Medicine, and Caring Sciences, Linköping University, Linköping, Sweden; 9 Novo Nordisk Center for Protein Research, Faculty of Health and Medical Sciences, 53139 University of Copenhagen, Copenhagen, Denmark; 10 Department of Health and Social Management, 122208 University of Eastern Finland and Wellbeing Services County of North Savo, Kuopio, Finland; 11 Department of Nursing Science, University of Turku and Research Services, Wellbeing Services County of Southwest, Turku, Finland

**Keywords:** Common data model, federated learning, data governance, delivery of health care, informatics, standardization

## Abstract

**Objective:**

This rapid review was undertaken to summarize contemporary knowledge on the application of common data models (CDMs) for semantic data standardization in the field of healthcare and provide a set of recommendations to guide the development of a CDM.

**Methods:**

The review adapted the Cochrane methodological recommendations for rapid reviews, namely (1) topic refinement, (2) setting eligibility criteria, (3) searching, (4) study selection, (5) data extraction, and (6) synthesis.

**Results:**

A total of 69 studies were included in the analysis. The analysis resulted in three interconnected layers covering (1) the federated network, (2) the iterative application process of a CDM, and (3) the data management process of each partner.

**Conclusion:**

Development and implementation of CDMs is a collaborative and iterative process, highly affected by the boundaries set by the individual federated learning partners, and the nature of their data. Interdisciplinary collaboration in application of CDMs for federated learning and data governance of health data is mandatory, with a call to increase domain expert involvement in data management.

## Introduction

The growing availability of health data provides promising and exciting opportunities to advance healthcare through digital health technologies.^1^ Appropriate health data governance practices can enhance data privacy and security, data management and linkage, data access management, and secondary data use outcomes.^2^ Data governance entails the strategic control and regulation of data management processes, including data authority, policies, used data standards, and procedures.^3^

The identification of internal and external factors tied to data governance is supported by the *information technology*–*governance of IT*–*governance of data* (ISO/IEC 38505-1:2017) standard.^4^ Adapted to digital health research, data governance strategies can increase the value, manage the costs, and complexities as well as ensure adherence to regulations and confidentiality issues related to healthcare data.^5^ In health practice, data governance promotes ethical and professional protocols to ensure confidential, secure, and reliable patient data management.^6^ Data governance policies can also be complemented to consider more complex issues such as data justice, promoting socially just health data use and collection.^7^

*Artificial intelligence* (AI) is an umbrella term to describe systems functioning with a level of autonomy, processing inputs and functions from humans and machines to achieve given objectives.^8^ They may use machine learning approaches to produce predictions, classifications, recommendations, decisions, and generative outputs to influence the environment in which the system operates.^8^ AI technologies provide ample opportunities to transform approaches related to patient care and administrative processes to better support healthcare provision.^9^ Combined with the massive amounts of patient data collected in healthcare, AI and particularly machine learning hold the potential to improve and speed up care processes, improve diagnostic accuracy, optimize the delivery and timing of treatments, and further improve care quality.^10–12^

*Sensitive data*. Using highly confidential and sensitive patient data for research purposes raises substantial ethical and legal questions and risks concerning the patient's privacy as well as data security, including cyber security.^13,14^ Furthermore, training AI models using data from single sources creates a risk of biased analysis outcomes.^15^ These biases can be harmful to the most vulnerable patient groups, exacerbating underlying inequalities stemming from patients’ social, ethnic, cultural, racial, or gender status.^16^ The necessity to collect diverse large-scale data sets to support sustainable AI model development is evident.^15^

*Federated learning* is a framework to guide decentralized research collaboration, predominantly machine learning. AI models are trained locally, sharing model updates instead of collecting and combining data sets, preserving the data privacy of individual participants.^17,18^ It presents a viable solution to enable development of powerful AI models and support large-scale AI-driven analytics within healthcare, increasing the potential to conduct impactful universal research on a data secure way.^17–19^ Federated learning in healthcare has historically focused on tasks relevant to medical fields such as radiology and internal medicine, with limited practical clinical applications.^20^

Benefits of federated learning approaches include a means toward preserving patient confidentiality and ensuring data security while providing a standard-based collaborative learning strategy.^21^ It appears to present a viable solution to support large-scale AI-driven analytics within healthcare, increasing the potential to conduct impactful universal research.^19^ However, issues related to varying data types between the federated learning partners need to be addressed to promote the development and training of AI-based models.^22^

*Health informatics standards* are officially approved documents, developed through consensus and evidence, setting rules, guidelines, or characteristics for activities and outcomes related to health information and communications technology.^6^ They address multiple aspects of data use and exchange, covering different functional layers and levels of abstraction. Structural standards, such as data exchange standards HL7 and FHIR^®^, are developed to promote interoperability and data sharing between different stakeholders and systems within healthcare ecosystems.^23^

Semantic standards, such as standardized terminologies, consist of standardized terms and definitions providing a unified, discipline-specific language that can be used for data entry, storage, and use.^24^ Systematized Nomenclature of Medicine Clinical Terms (SNOMED CT) is a globally recognized and widely adopted standardized terminology, covering a broad range of health-related topics, with concepts including clinical findings, procedures, body structures, social contexts, and clinical qualifiers.^25,26^ Another widely used standardized clinical terminology, Logical Observation Identifiers Names and Codes (LOINC), provides codes for health measurements, observations, and documentation.^27^

Compared to standardized terminologies, standardized classification systems present broader categories for data organization, such as the International Classification of Diseases (ICD)^24^ and the Anatomical Therapeutic Chemical (ATC) classification to classify pharmacological substances.^28^ Despite the continuous work conducted in the field of data governance, the systems, customs, structures, and terminologies used in healthcare documentation vary between institutions and geographical locations.^29^

*Common data models* (CDMs) have been developed to facilitate the utilization of heterogeneous data sources in large-scale collaborative research.^30^ They are frameworks to consistently organize and store data from different sources into large, standardized data storages, promoting cross-institutional collaboration.^30^ Data standardization is the process of converting data from different sources to a common format to promote data sharing.^31^ It is commonly carried out using the extract, transform, and load (ETL) process that captures both the structure and the semantics of the data.^32^ The ETL process comprises methods used to retrieve source data (extract), convert the data to a standardized format (transform), and integrate the transformed data to the target data repository (load).^33^ Semantic models are used to standardize various clinical observations and other patient data needed to conduct analytical tasks in health research.^34^ In federated learning, CDMs are implemented to enable sharing analytical codes between institutions without a need for model modifications.^35^

The Observational Health Data Sciences and Informatics (OHDSI) community is an ample example of an active multinational research collaboration to facilitate large-scale data analytics. The Observational Medical Outcomes Partnership (OMOP) CDM, managed by the OHDSI group, is developed to standardize observational healthcare data in structured and free-text formats.^36^ OMOP CDM has been widely adopted in health research for the standardization of data to support AI development, especially within machine learning and natural language processing.^37^ SNOMED CT and LOINC are the most notable health informatics standards incorporated into the OHDSI vocabularies used in the OMOP CDM.^36^

The aim of this rapid review was in particular to summarize present contemporary knowledge on the application of CDMs for semantic data standardization in the field of healthcare and provide a set of recommendations to guide the development and utilization of CDMs in AI technology development.

## Materials and methods

**Figure 1.**
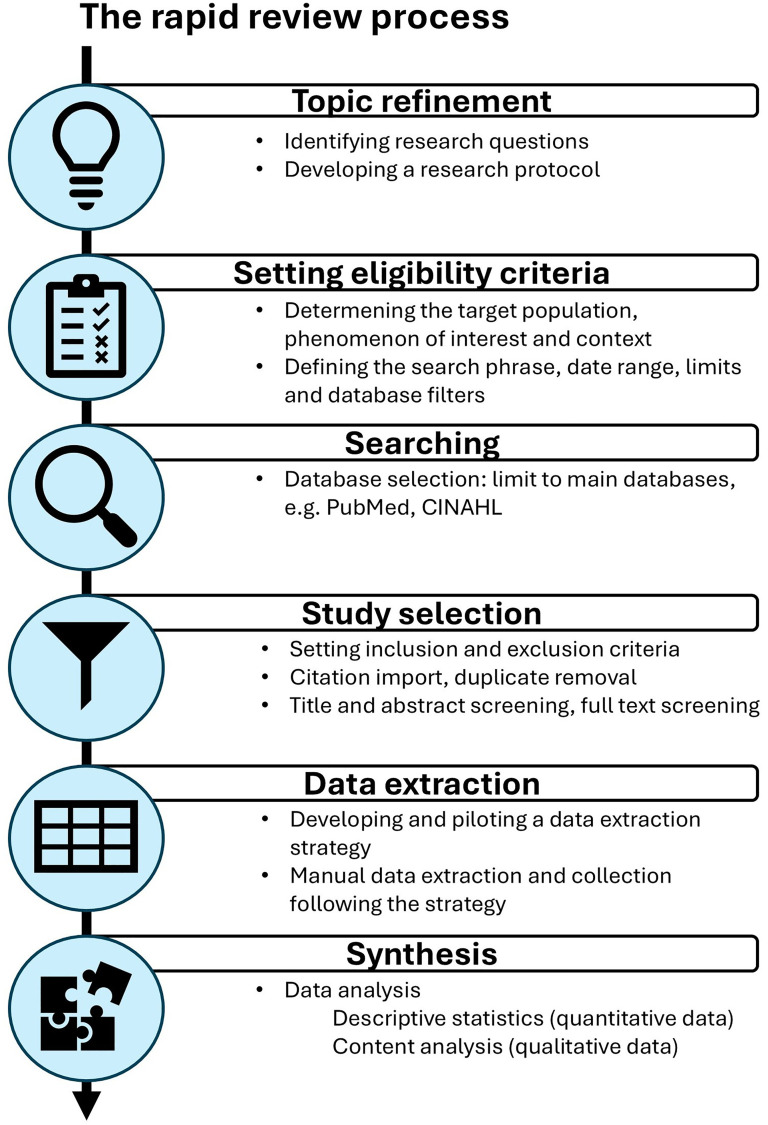
Overview of the rapid review process following the Cochrane rapid reviews methods group recommendations.

A rapid review was undertaken to efficiently summarize the current knowledge regarding the implementation of CDMs for semantic data standardization in healthcare research to support the CDM development for a federated learning project.^38^ Rapid reviews are a streamlined approach of knowledge synthesis, modifying and accelerating the process of systematic reviews to provide timely results to support decision making.^39^ The review adapted the Cochrane methodological recommendations proposed by Garritty et al., namely (1) topic refinement, (2) setting eligibility criteria, (3) searching, (4) study selection, (5) data extraction, and (6) synthesis, as presented in Figure 1.^39^ The PRISMA guideline for reporting systematic reviews was adapted to report the results.^40^

### Topic refinement

The topic refinement included identifying the research questions and developing a review protocol.^39^ The review was set to answer the following research questions:

1. What elements have been reported on the development and use of CDMs when dealing with health data?
2. What considerations need to be undertaken when implementing CDMs in the development of AI-based technologies for healthcare?

A protocol was published on the OSF registries (https://osf.io) on 17.1.2024, and it can be accessed through https://doi.org/10.17605/OSF.IO/YJGBS.

### Setting eligibility criteria

The eligibility criteria were set using the “PICo strategy” used commonly for qualitative reviews, with the core elements including Population (P), phenomenon of Interest (I), and Context (Co).^41^ The following PICo strategy was constructed within the research team to define the search phrase:

Population = all healthcare users, all healthcare settings
Intervention = semantic data standardization using a CDM
Context = all healthcare contexts, textual real-world health data

### Searching

The literature search was conducted in August 2024 using the PubMed (MEDLINE) and CINAHL (Ebsco) databases that comprehensively cover biomedical and health sciences literature. No explicit date range was set, and all historic studies accessible in the databases that were published by August 6, 2024, were admitted.

To comply with the PICo strategy, the search was conducted using the search phrase “Common data model” OR ((“data harmoni*” OR “data standard*” OR “data model” OR “data interoper*”) AND (“federated learning” OR “distributed machine” OR “distributed learning” OR “decentralized learning” OR “decentralised learning” OR “collaborative learning”)) in both databases. No applicable MeSH and CINAHL subject headings were available or added to the search phrase. No filters or refinements provided by the databases were used, including but not limited to filters for the text availability, article attribute, article or source type, publication date, or article language.

### Study selection

All peer reviewed original study designs describing the process of standardizing textual data from real-world healthcare settings using a CDM were included in the study. No exclusions were made regarding the study participants or setting. Only articles written in English were included. Studies were excluded, if they did not contain a clear description of the process of standardizing and transforming textual real-world health data using a CDM. For this review, health data was defined as any patient-level data created by health professionals within a healthcare system. This data includes, but is not limited to, electronic health record (EHR) data and administrative data containing patient information, observations, or outcomes.

Identified article titles and abstracts were downloaded into the Rayyan web application (www.rayyan.ai) for title and abstract screening. Duplicates were identified using the automatic detection provided by Rayyan and removed manually by comparing the identified titles and deleting the confirmed duplicates by one researcher. Two researchers then independently and manually reviewed the titles and abstracts, followed by full-text screening. The title, abstract, and full-text screenings were conducted using the “Blind On” function provided by the Rayyan web application, where the decisions, labels, or notes made by individual researchers are not visible to the others. All contradicting screening results related to inclusion or exclusion were discussed between the reviewers to reach a consensus.

### Data extraction

A data extraction strategy was created to extract the relevant information from the included studies. The strategy was tested and refined using a sample (*n* = 10) of the included full-text articles. The following data elements were extracted and collected manually by one researcher from all the included articles using the Webropol Survey & Reporting application (webropol.com):

- Manuscript details (authors, year of publication, countries, aim of the study, and setting)
- Source data details (data source, sample size, data type, and data elements)
- Clinical coding systems used in source and target data
- Phases of the data standardization process and considerations
- Clearly discussed elements of data governance
- Considerations regarding the application of CDMs in federated learning tasks

### Synthesis

The data were downloaded into a spreadsheet. Quantitative data were calculated manually and analyzed using descriptive analysis, and qualitative data with the content analysis methods.^42^ The elements of data governance were analyzed deductively, using the good data governance practices checklist for real-world health data (adopted from Solà-Morales et al.).^2^ To evaluate the acceptability, quality, and integrity of data governance practices, the elements of data governance have been divided into four sections, namely

1. Data privacy and security, including patient consent, data de-identification, anonymization, or pseudonymization
2. Data management and linkage, including data standardization, source data quality, consistency, accuracy and completeness, and possible data bias
3. Data access management, including ethical or institutional review board grant for data access
4. Generation and use of real-world evidence, including minimum quality criteria for the data for secondary use purposes

## Results

### Overview of the included studies

**Figure 2.**
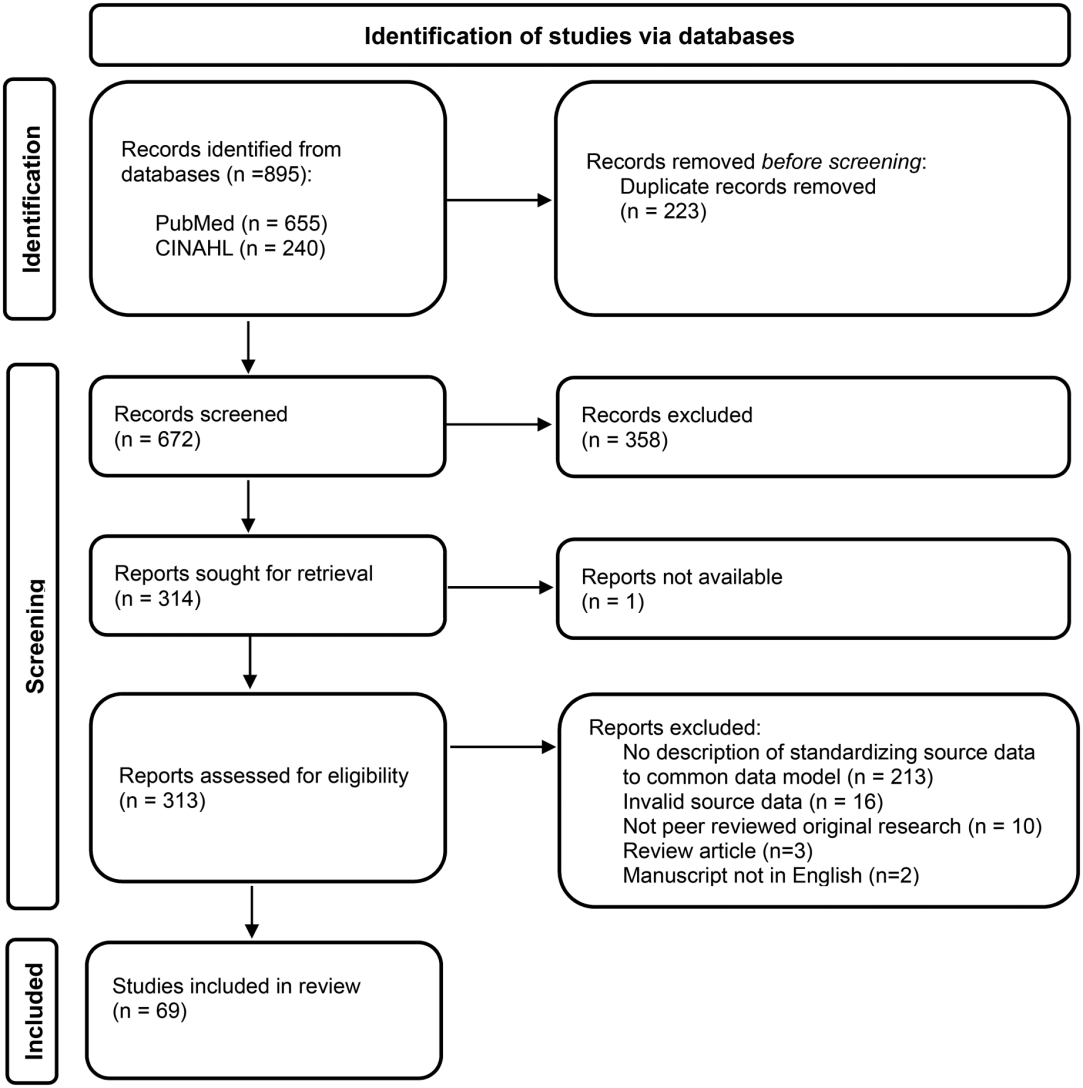
PRISMA flowchart diagram of the article screening. Adapted from Page et al.^40^

A total of 890 manuscript titles were retrieved based on the database searches for this study. After removing the duplicates, 672 unique entries were included in the title and abstract screen, as illustrated in the PRISMA flowchart diagram in Figure 2. A total of 314 full-text manuscripts were screened, resulting in 69 original studies included in this review, after applying the exclusion criteria as described in Figure 2.

**Figure 3.**
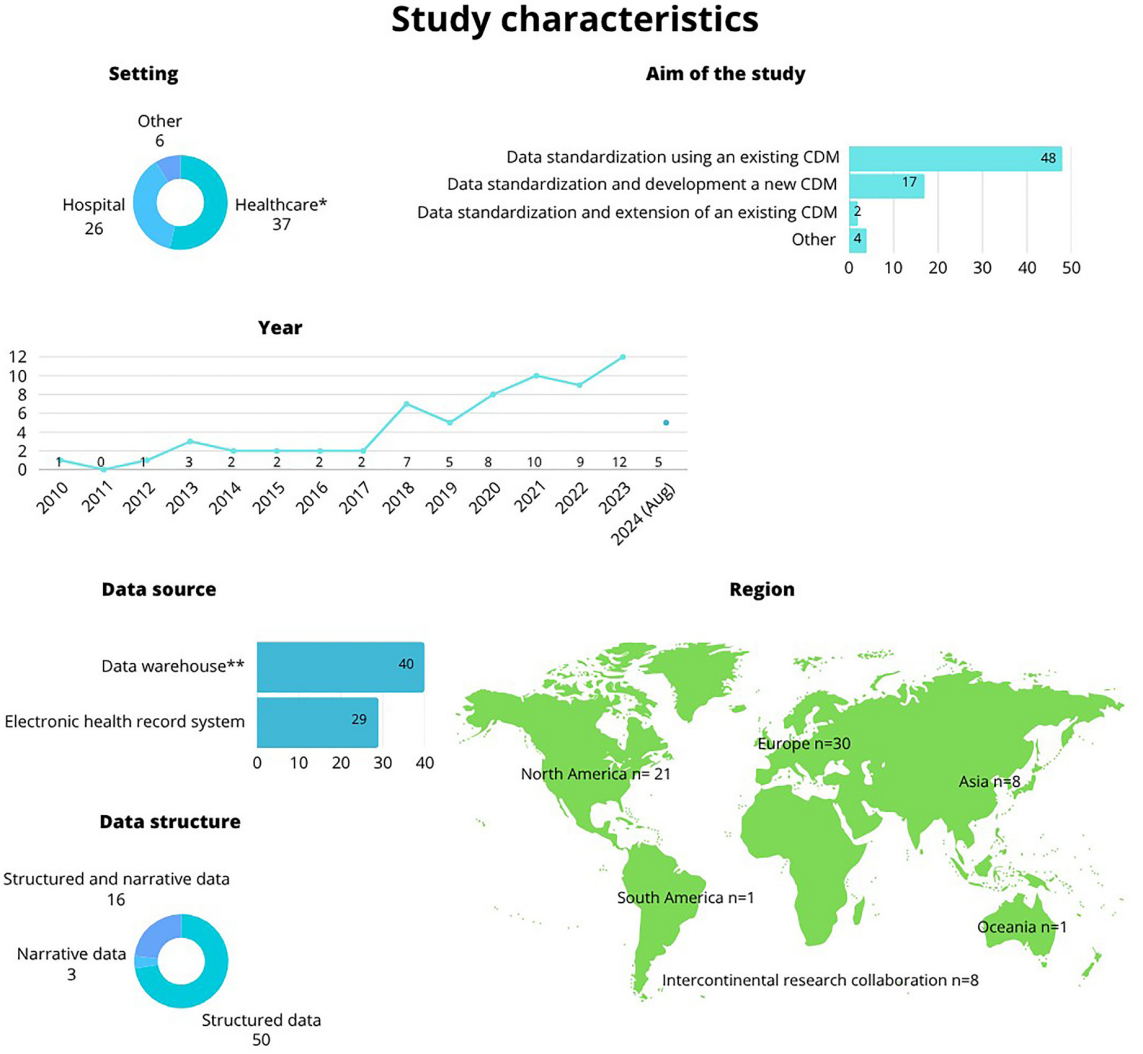
Characteristics of the admitted studies. *In setting: healthcare includes data combined from various different healthcare environments not explicitly defined by the authors, other include, for example, skilled nursing facilities and memory clinics. **In data sources: data warehouse includes data from multiple sources such as electronic health records, health data registries, and biobanks.

Nearly half (*n* = 30, 43.5%) of the studies were conducted in Europe, followed by North America (*n* = 21, 30.4%), Asia (*n* = 8, 11.6%), South America (*n* = 1, 1.4%), and Oceania (*n* = 1, 1.4%), with eight (11.6%) studies conducted as collaborative intercontinental research, as described in Figure 3.^21,^^43–110^ The studies were published between 2010 and 2024, with more than half (*n* = 36, 52.2%) of them published after 2020.

The aim of the studies was predominantly to standardize data by applying an existing CDM (*n* = 48, 69.6%). Over half of the studies used data that was derived from different healthcare settings (*n* = 37, 53.6%) that were not explicitly described by the authors. Over half of the data (*n* = 40, 58.0%) used in the studies were stored in data warehouses that included multiple data sources, such as EHRs, health data registries, and biobanks, using mostly structured data (*n* = 50, 72.5%). The number of patients in the datasets used was reported in 43 (62.0%) studies, with numbers ranging from 100 to 88 million patients, with an average of 4.3 million patients.

The data extraction results are presented in full in the Supplemental materials.

**Figure 4.**
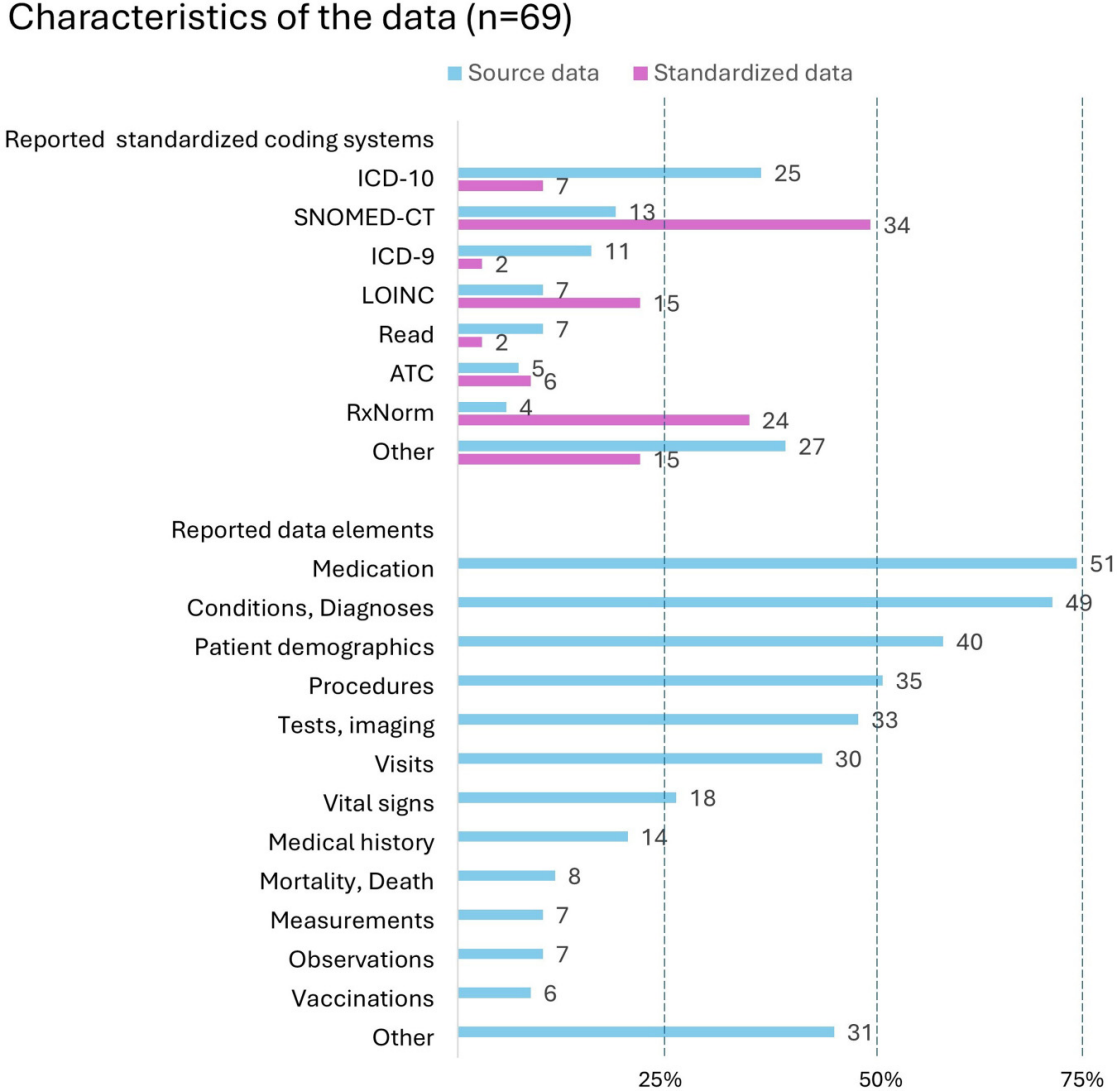
Reported standardized coding systems and data elements used in the reviewed articles (*n* = 69) for source and standardized data.

The most used standardized coding systems reported in the source data were ICD-10 (*n* = 25, 36.2%), SNOMED CT (*n* = 13, 18.8%), and ICD-9 (*n* = 11, 15.9%), as presented in Figure 4. The standardized data elements were commonly related to medication and prescriptions (*n* = 51, 73.9%) followed by conditions and diagnoses (*n* = 49, 71.0%), procedures (*n* = 35, 50.7%), and patient demographics (*n* = 40, 58.0%). Other data elements (*n* = 31, 44.9%) included a variety of clinical data, symptoms, assessments, and risk factors. These elements were predominantly standardized using SNOMED CT (*n* = 34, 49.3%), RxNorm (*n* = 24, 34.8%), and LOINC (*n* = 15, 21.7%) in the CDMs.

### CDM adoption in federated learning projects

**Figure 5.**
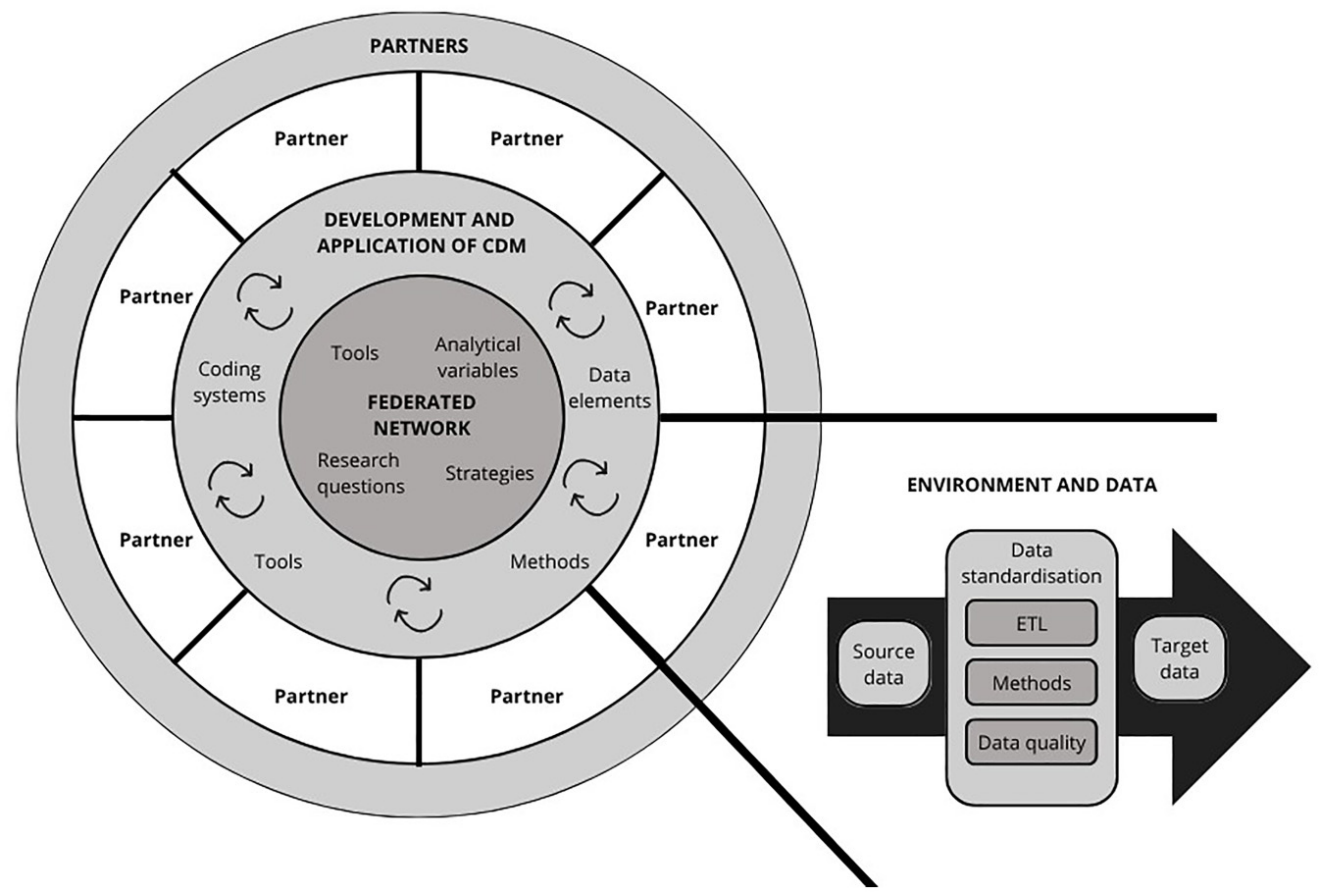
Overview of the elements related to development and application of common data models (CDM) in federated learning projects, where ETL represents the “extract, transform, and load” process and arrows indicate an iterative development process highlighting the importance of communication and collaborative work between partners in the federated network for the targeted CDM.

The elements related to the development and implementation of CDMs in federated learning projects were parted into three categories: (1) the federated network, (2) the development and application of CDM, and (3) the federated learning partners, as visualized in Figure 5. To facilitate successful data standardization, communication and collaboration were perceived as key in the reviewed studies.

#### The federated network

The tools, strategies, analytical variables, and research questions steering the implementation of a CDM under development were discussed within the network and refined based on the feedback provided by the partners. Communication between the project partners and interdisciplinary working teams was portrayed as a key element to facilitate successful collaboration in the reviewed studies.^44,53,106^ It was important that the partners shared an understanding of the research questions and roles and responsibilities for each participant.^21,62^ This included exchanging knowledge and understanding the differences between the research datasets.^107,108^ These differences could include a variation in used coding systems, terminologies, data elements, data structures, and even languages.^44,45,78,93,107,108^ CDMs were perceived as a facilitator for multicenter research to overcome the issues regarding heterogeneity of the source data, but their deployment required careful and consistent planning regarding, for example, clearly defined variables and data elements needed for the analysis as well as the tools used in data standardization.^44,46,74,93^^104–106^

#### Development and application of CDMs

Development and application of CDMs was driven by the needs of federated networks while also considering possibilities and constraints of the federated learning partners regarding the used coding systems, methods, and required data elements. In the reviewed studies, the majority (*n* = 51, 73.9%) applied or extended an existing CDM to standardize raw data, predominantly the OMOP CDM (*n* = 43, 62.3%). The choice of a CDM was guided by the use case of the target data and basic data elements needed for the data analysis.^44,51,54,57,62,70,80,86,93,99,104,108^ If an existing CDM was considered inadequate to meet the needs of the project, it could be extended through the implementation of new concepts or vocabularies.^49,54,55,57,62,64,72,97^

A study-specific CDM might result in less data loss than using a standard CDM.^55,78^ In total, 18 (26.1%) studies sought to resolve this issue by developing a novel CDM. A successful CDM development process required collaboration between domain experts, information system architects, and researchers.^50,75^ The key data elements, relevant ontologies, and standards were identified through expert consultations, publicly available data, established procedures, or various clinical indicators.^43,48,75,77,78,86,89^

#### The federated learning partners

Individual partners within the federated network were responsible for creating suitable environments as well as providing the data to facilitate data standardization. All partners were required to have access to hardware that fulfilled the memory, space, and performance requirements needed to run the processes.^44,76^ The information technology infrastructure was required to promote data extraction, posing demands for the project-specific software and tools used for data storage and preparation.^44,55,77,101^

The standardization process was predominantly (*n* = 49, 71.0%) explained through the ETL process, in which the source data was retrieved from the EHR or database, standardized to the CDM format, and loaded into a data repository. ETL was usually (*n* = 52, 75.4%) accompanied by various concept mapping methods. The standardization process was guided by the source data as well as the target CDM, combining automated and manual methods.

Finally, data quality and characterization checks were important to ensure the completeness and validity of the standardization process.^43,45,48,53,55,65,71,73,94,96,101,106^

### Data governance in CDM development and federated learning

**Figure 6.**
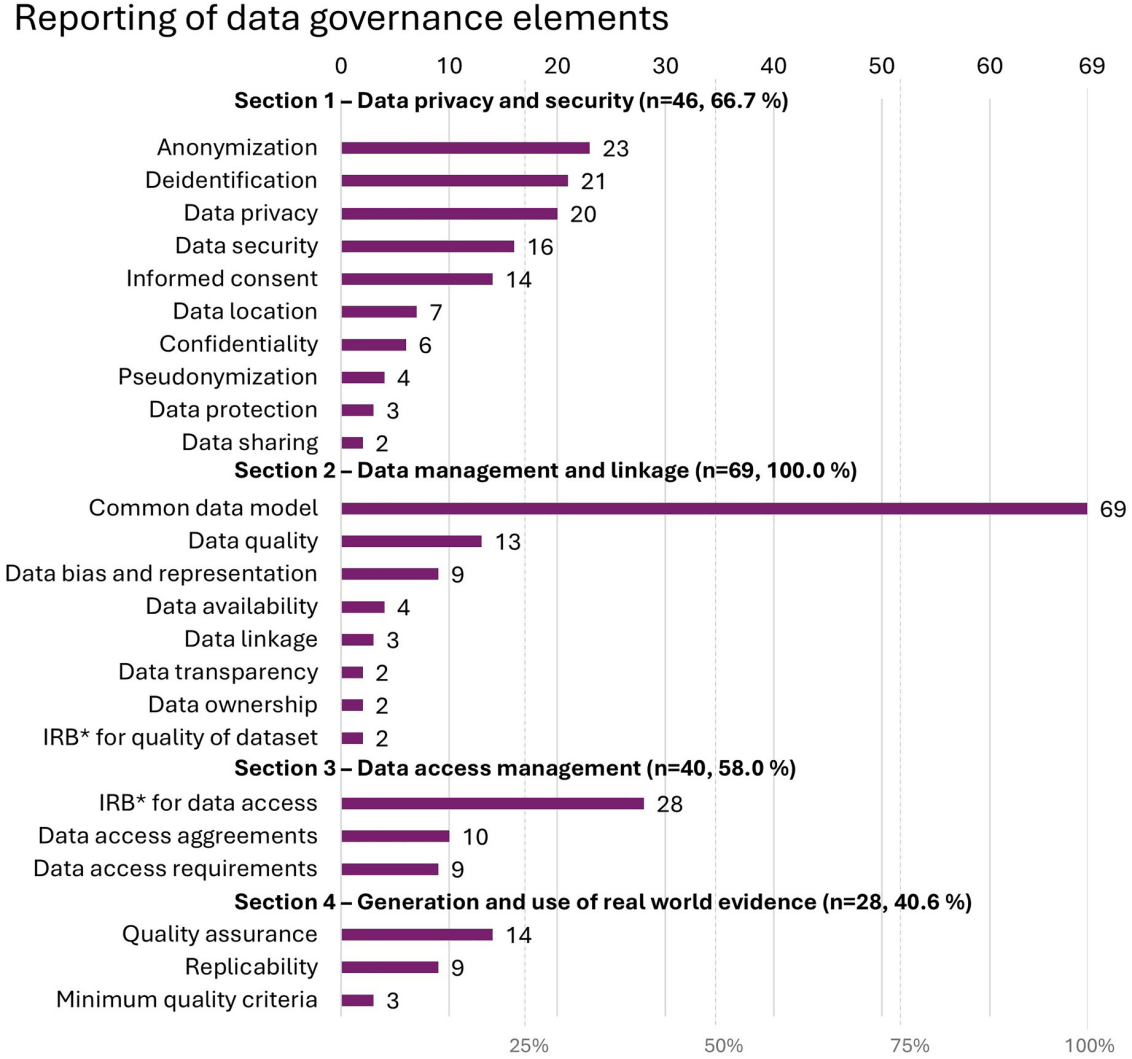
Reporting of data governance elements guided by the data governance checklist, where IRB* refers to the institutional or ethical review boards appraising the compliance of the research with ethical and regulatory standards.

A total of 13 (18.8%) of the included studies mentioned the concept of data governance. When looking at individual categories of the data governance checklist (see Materials and methods), practices related to *section 1—data privacy and security* were discussed in 46 studies (66.7%), as presented in Figure 6. These included addressing methods to safeguard patient identity through anonymization (*n* = 23) meaning permanent removal of all identifiable information, pseudonymization (*n* = 4) meaning replacement of identifiable information with pseudonyms or codes, and de-identification (*n* = 21), which can include either or both aforementioned methods. Data protection measures included discussion related to data privacy (*n* = 20), security (*n* = 17), and confidentiality (*n* = 6), whereas participant rights were discussed addressing informed consent (*n* = 14).

As data standardization using a CDM was one aspect of data governance in *section 2—data management and linkage*, all studies (*n* = 69, 100%) touched on subjects in this category. Other practices discussed included data quality (*n* = 13) in terms of data consistency, precision, coverage, and timeliness, followed by discussion related to data bias and representation (*n* = 9). The practices covering *section 3—data access management* were mentioned in 40 (58.0%) studies, mainly disclosing the approval for data use as reviewed by institutional or ethical review boards (*n* = 28).

Finally, practices related to *section 4—generation and use of real-world evidence* were referred to in 28 (40.6%) studies, as the quality assurance (*n* = 14) and replicability (*n* = 9) practices were discussed for future evidence creation.

## Discussion

This rapid review presented the growing body of knowledge on standardizing health data using established and novel CDMs from a federated learning perspective. The most common feature we found was the iterative nature of the process which was highlighted through continuous collaborative efforts when developing compatible, adaptable, and user-friendly solutions. While recognizing the sensitivity and privacy concerns related to using real-world health data, the issue of data governance has received limited attention in research surrounding CDM applications.

The results of this review underline the importance of acknowledging not only the possibilities provided by the utilization of standardized data in large-scale federated learning projects, but also the boundaries set by the individual partners regarding the source data as well as the resources to conduct the standardization process. This is in line with previous literature on federated learning projects aiming to standardize data using an established CDM. Participants of the European Health Data & Evidence Network project highlighted the significance of multiprofessional teams with expert knowledge on the source data, the CDM, and the implementation of the process to facilitate successful data standardization.^111^ The choice of CDM should be guided by its suitability for the intended use, resulting in a need to carefully inspect its completeness, simplicity, integration, and implementability for the project.^112^

Issues related to data quality were reported in some of the reviewed articles, underlining the institutional and national diversities of data governance policies and the associated complications. Previous studies have reported the dual nature of data governance in big data research, which is predominantly defined by over- or underregulation, calling for interdisciplinary roadmap development to support efficient data governance policies.^113^ In the European Union, relevant directives to regulate data use in AI development include the General Data Protection Regulation, the European Health Data Space, the Artificial Intelligence Act, and the Medical Device Regulation.^114^ While the key elements of data governance have been adopted in most economies, it has been reported that local governments seldom utilize collected feedback in revising the legislation.^115^ The need to develop a global framework and a golden standard for data governance and reporting in healthcare through interdisciplinary collaboration is evident to ensure ethical, consistent, and accurate data storage and use in future technology development.

The second issue related to data quality is closely tied to the healthcare professionals’ competencies and contextual understanding of documentation practices, data models, and system logic. Commonly used data structures and standards should serve a broad range of users within the healthcare domain, including physicians and nurses, affecting the primary and secondary use of generated health data. Future research is needed to investigate the AI literacy of healthcare professionals and means to develop their readiness to engage in such projects. Previous research investigating quality of healthcare documentation suggests a need to revise current documentation practices to ensure the quality, conformity, usability, and readiness of health data for secondary purposes.^116^ Comprehensive guidelines to increase the understanding and guide the use of different health informatics standards, complemented with tools to evaluate the quality of documentation, are needed to ensure the completeness and cohesion of healthcare data to comply with the technical requirements related to sustainable, safe, and ethical AI development. Increasing commitment and engagement with these guidelines as well as novel technologies through educational interventions and support from healthcare management will result in increased work efficiency, more reliable AI tools, and safer patient care.

Limitations to this study concern the nature of the rapid review, as only two (albeit the most widely used) databases were searched. Moreover, the included articles were not assessed for their scientific quality. Additionally, only articles written in English were admitted to this study, which may contribute to a bias.

Current research literature acknowledges the lack of comprehensive methodological frameworks to guide CDM development.^30^ Additional and repeated research efforts are warranted to refine methodological principles to streamline CDM development and data standardization in healthcare research. Furthermore, reporting guidelines on CDM development and implementation are needed to certify adequate transfer of knowledge.

## Conclusions

This rapid review summarizes current knowledge on the development and applications of CDMs in healthcare with a particular perspective on supporting the development and implementation of AI technologies in federated learning. Our findings

1. emphasize the essential role of interdisciplinary collaboration in the iterative process of development and application of CDMs for federated learning in healthcare and
2. highlight the importance of developing unified data governance policies to ensure safe and reliable AI development, with an urgent call to increase domain expert involvement in data management.

Healthcare professionals representing a variety of domain knowledge should actively seek engagement in interprofessional collaborations. This is deemed to increase the number of healthcare professionals with sufficient competencies, skills, knowledge, and motivation to facilitate the development of AI and the secondary use of health data, but also on interdisciplinary teamwork. The competencies and knowledge possessed by information technology professionals and researchers is vital to ensure functioning technological infrastructures and facilitate the implementation of necessary tools. Likewise, stakeholder, medical, and health professional knowledge is the key in understanding the data elements, concepts, and vocabularies as well as the clinical needs and favorable outcomes. Further, health informatics specialists representing different health domains are needed to promote a common understanding within the team, as well as enhance the ethical and regulatory knowledge guiding the process. It requires systematic strategic leadership to facilitate the organizational and national infrastructures that promote participation and motivation in interprofessional research and development initiatives. It would be of particular interest to investigate the AI literacy of health professionals and means to develop their readiness to engage in such projects, with the knowledge and the ability to engage in discussions and understand the complexities related to large-scale AI development. More education and guidance to enhance and tools to evaluate the quality of documentation are needed.

## Supplemental Material

sj-xlsx-1-dhj-10.1177_20552076251395536 - Supplemental material for A rapid review on the application of common data models in healthcare: Recommendations for data governance and federated learning in artificial intelligence development Supplemental material, sj-xlsx-1-dhj-10.1177_20552076251395536 for A rapid review on the application of common data models in healthcare: Recommendations for data governance and federated learning in artificial intelligence development by Hanna von Gerich, Taridzo Chomutare, Ville Kytö, Peter Lundberg, Troels Siggaard and Laura-Maria Peltonen in DIGITAL HEALTH
